# Exploiting *in vitro* potential and characterization of surface modified Zinc oxide nanoparticles of *Isodon rugosus* extract: Their clinical potential towards HepG2 cell line and human pathogenic bacteria

**DOI:** 10.17179/excli2018-1327

**Published:** 2018-07-16

**Authors:** Aisha Siddiquah, Syed Salman Hashmi, Sadaf Mushtaq, Sullivan Renouard, Jean Philippe Blondeau, Rashda Abbasi, Christophe Hano, Bilal Haider Abbasi

**Affiliations:** 1 Department of Biotechnology, Quaid-i-Azam University, Islamabad-45320, Pakistan; 2 Laboratoire de Biologie des Ligneux et des Grandes Cultures (LBLGC), UPRES EA 1207, Université d'Orléans, Chartres, France; 3 Conditions Extrêmes et Matériaux: Haute Température et Irradiation (CEMHTI) CNRS UPR3079, 1D avenue de la Recherche Scientifique, 45071 Orléans, France; 4 Institute of Biomedical & Genetic Engineering (IBGE), Sector G-9/1, Islamabad, Pakistan

**Keywords:** nanotoxicity, ZnONP, pH, surface adaptation, characterization, Isodon rugosus

## Abstract

Little is known about biogenically synthesized Zinc oxide nanoparticles (ZnONPs) from *Isodon rugosus*. Synthesis of metal oxide NPs from essential oil producing medicinal plants results in less harmful side effects to the human population as compared to chemically synthesized NPs. In this article, we report biogenic synthesis of ZnONPs from *in vitro* derived plantlets and thidiazuron (TDZ) induced callus culture of *Isodon rugosus*. Synthesized NPs were characterized using UV-spectra, XRD, FTIR, SEM and EDX. Furthermore, the NPs were evaluated for their potential cytotoxic (against HepG2 cell line) and antimicrobial (against drug resistant *Staphylococcus epidermidis*, *Bacillus subtilis*, *Klebsiella pneumoniae* and *Pseudomonas aeruginosa*) activities. Pure crystalline ZnONPs with hexagonal and triangular shapes were obtained as a result of callus extract (CE) and whole plant extract (WPE), respectively. ZnONPs showed potent cytotoxic and antimicrobial potential. The antimicrobial and cytotoxic activities of ZnONPs were found to be shape and surface bound phytochemicals dependent. CE mediated hexagonal ZnONPs showed superior anti-cancer and antimicrobial activities as compared to WPE mediated triangular shaped ZnONPs. It is concluded that biogenic ZnONPs have incredible potential as theranostic agents and can be adopted as useful drug delivery system in next generation treatment strategies.

## Abbreviations

ZnONPs: Zinc oxide nanoparticles, CE: Callus extract, WPE: Whole plant extract, C-ZnONPs: Callus derived zinc oxide nanoparticles, W-ZnONPs: Whole plant derived zinc oxide nanoparticles, MS: Murashige and Skoog medium, TDZ: Thidiazuron, TPC: Total phenolic content, TFC: Total flavonoid content, DW: Dry weight, PGRs: Plant growth regulators, UV-vis: Ultra violet-visible, FTIR: Fourier-transform infrared, XRD: X- ray diffraction, SEM: Scanning electron microscopy, EDX: Energy dispersive X-rays, HepG2: Human hepatocellular carcinoma cell line, DMEM: Dulbecco's Modified Eagle's medium, SRB: Sulforhodamine B, TCA: Trichloroacetic acid, ROS: Reactive oxygen species, DMSO: Dimethyl sulfoxide, CFU: Colony forming unit 

## Introduction

In recent years, chemotherapy, radiation and surgery are among the main strategies to treat cancer. The problem with such strategies is they are highly uneconomical. Production of nanomedicine through green route is an economic alternative to the aforementioned strategies (Gowda et al., 2014[16]). Plant extracts have the potential to produce nanoparticles (NPs) having specific size, shape, and composition. Green nano-biotechnology utilizes the reducing and stabilizing agents present in the plant extracts through an alternative bottom-up approach that results in formulation of stable and biocompatible NPs (Parveen et al., 2016[35]). Bio-synthesis of NPs follows three main phases: selection of suitable solvent, a benign and eco-friendly reducing agent and nontoxic capping agent. Alteration in the aforementioned agents results in controlled synthesis for preparation of NPs with specific morphological features. Controlled synthesis of zinc oxide (ZnO) NPs with unique features like selectivity, cell imaging, bio-sensing, drug/gene delivery and enhanced cytotoxicity may serve as an alternate anticancer strategy (Lewinski et al., 2008[29]; Sur et al., 2010[48]). ZnONPs have been categorized as “GRAS” (generally recognized as safe) substance by FDA owing to their biocompatible nature, thus, making them applicable for drug delivery (Jamdagni et al., 2016[24]). ZnONPs possess properties of semiconductors due to their components (Zn and O) which belong to d-block (2^nd^ group) and p-block (6^th^ group) of the periodic table, respectively. The logic for this is that ZnONPs have high band gap (3.37 eV) and exciton binding energy (60 meV) (Neumark et al., 2007[34]). Therefore, it can tolerate detectable electric fields, adequate temperatures, and high power activities.

The efficacy of ZnONPs has been evaluated both independently and in complexes with organic compounds. Conjunction of NPs with cells biologically activates the cellular proteins to minimize the toxicity (Abbasi et al., 2017[1]; Riaz et al., 2018[42]; Balaji and Gothandam, 2016[9]; Huang et al., 2007[21]). The generation of cellular reactive oxygen species (OH^∙^, O_2_^∙^, HO_2_^∙^) from the surface of ZnONPs is believed to be a major component of its cytotoxicity, yet the mechanism of cytotoxicity is not fully understood. Overproduction of these highly active free radicals has been shown to instigate oxidative stress in macrophages which in turn elicit apoptosis due to insufficient anti-oxidative protective mechanism (Willet, 2002[54]). 

Previous studies investigated the *in vitro* cytotoxicity of ZnONPs by exploiting variety of mammalian cell lines (Xia et al., 2008[55]; Yuan et al., 2010[56]; Heng et al., 2011[19]). Moreover, the impact of shape and size of ZnONPs on their cytotoxic potential has also been explored. Nagarajan and Kuppusamy (2013[32]) studied ZnONPs with different shapes (hexagonal and triangular) regardless of their specific surface area. Practically, variations in capping agents (secondary metabolites) bound to the surface of NPs, influence their physiochemical action within cell culture media resulting in different levels of cytotoxicity (Bogutska et al., 2013[10]). Alternatively, surface charge ratio of NPs also play considerable role in cytotoxicity, as positively charged NPs were shown to possess a higher cytotoxicity. Outer layer of the cancer cell holds negatively charged phospholipids (Abercrombie and Ambrose, 1962[3]). The electrostatic interaction between positively charged ZnONPs and negatively charged phospholipids on cell surface affects cellular uptake and intracellular localization of ZnONPs (Rasmussen et al., 2010[41]; Sultana et al., 2017[47]).

Hepatocellular carcinoma (HCC) is the fifth most common malignant tumor, with a poor survival rate of 10 % (overall 5 years). HepG2 (Human hepatocellular carcinoma) cell line has been used extensively to study HCC and drug metabolism due to its phenotypic stability (Hanley et al., 2008[18]). Shelf-life of conventional single drug moiety is usually limited to few minutes and requires repeated injections (Wang et al., 2009[52]). However, capping of NPs strengthen shelf-life of the drugs up to several hours, thus increasing their availability to target cancer cells. The objective of the current study was to assess the *in vitro* cytotoxic potency of biologically synthesized hexagonal and triangular ZnONPs against HepG2 cell line and drug resistant bacteria.

## Material and Methods

### Preparation of Isodon rugosus plant extract

**Figure 1:**
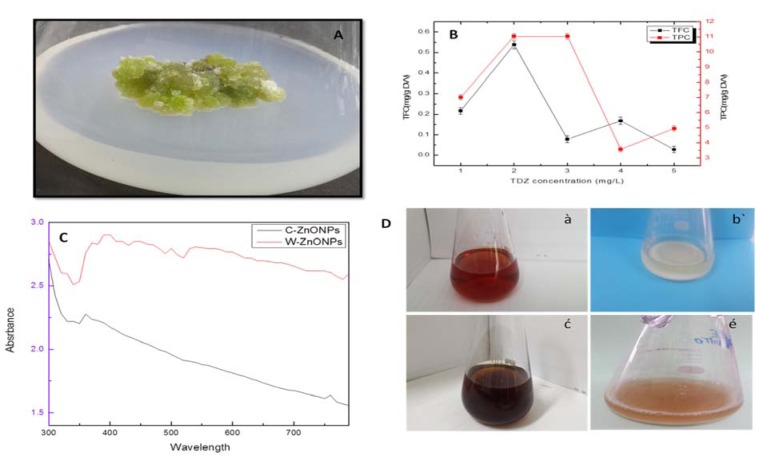
(A) Thidiazuron induced callus from stem explant, (B) Estimation of phenolic and flavonoid content in callus extract, (C) UV-Vis spectral analysis of ZnONPs, (D) Optical observation of biogenically synthesized ZnONPs using *Isodon rugosus* extract, (à) Thidiazuron induced callus extract, (bʹ) reaction mixture of callus derived ZnONPs, (ć) plant extract, (é) reaction mixture of plant derived ZnONPs. After completion of the reaction, presence of white precipitate indicates the synthesis of ZnONPs.

*Isodon rugosus *seeds, acquired from the Plant Cell Culture Laboratory, Department of Biotechnology, Quaid-e-Azam University, Islamabad, were germinated under sterile conditions. Callus culture was established on MS medium (Murashige and Skoog, 1962[31]) and maintained in growth room at 25±2 °C for 16/8 hrs (light/dark) photoperiod. After 40 days, explants fragments were shifted to MS medium supplied with various concentration of TDZ (1, 2, 3, 4, 5 mg/l) for callus induction (Figure 1A(Fig. 1)). Extract of Callus (CE) and *in vitro* derived whole plant (WPE) was prepared by adding 10 g of freshly harvested callus and plant sample separately into 500ml flask. 100 ml of distilled water was added to each flask and boiled. After overnight incubation at 40 °C, the broth was filtered carefully. These extracts were further used for the bio-reduction of Zn^+^ to Zn^°^.

### Biological synthesis of ZnONPs

Synthesis of NPs depends on type and concentration of plant extract, metallic salt, response time, pH and temperature (Dwivedi and Gopal, 2011[13]). Zinc Acetate Dihydrate (C_4_H_6_O_4_Z_n_) was used as a precursor solvent. 0.02 M solution of zinc acetate (50 ml) was mixed with 1 ml sample extract separately (1:50) and was stirred constantly. pH of the medium was maintained at 12 by adding 2M Sodium Hydroxide (NaOH) drop-wise. The reduction was attributed to phenolics, terpenoids, polysaccharides and flavones present in the extract. White and pale precipitates were seen for CE and WPE, respectively. The resulting precipitates were washed three times (first with ethanol and then twice with distilled water). Samples were centrifuged at 6000 rpm for 15 min and incubated overnight at 60 °C for drying. The powdered samples of ZnONPs obtained after drying were stored until further use.

### Detection of phytochemicals

The Folin-Ciocalteu reagent (FCR) method, also called the gallic acid equivalence method (GAE), was used to measure the total phenolic content in CE and WPE (Singleton et al., 1999[45]). This reagent does not only measure phenolic content but is also used to measure any reducing substance in the sample extract. Folin's reagent assist to detect amine and sulfur containing compounds, which are mainly involved in reduction and stabilization of NPs. In brief, 20 µl of sample extract (CE and WPE) was mixed with 90 µl of FC reagent. After 5 min of incubation, sodium carbonate (Na_2_CO_3_, 6 % w/v) was added. Absorbance was measured (in triplicates) at 725 nm (Huang et al., 2007[21]). 

To measure the level of flavonoids in CE and WPE, we followed the Willet's method of estimation with some alteration. 20 µl aliquot of sample extract was mixed with 10 µl of potassium acetate (1 M) and 10 µl of 10 % Aluminium chloride (AlCl_3_). Samples were incubated for 30 min, followed by addition of 160 µl distilled water. Absorbance was detected at 415 nm (Thermo Fisher Scientific, model 4001/4). Quercetin was used to make the calibration curve. Calculation of TFC was carried out in triplicate (Willet, 2002[54]).

### Structural and optical characterization of ZnO NPs

Characterization of the synthesized NPs for their configuration, diameter and surface modification was performed by XRD, EDX and SEM analysis. Optical properties of ZnONPs were characterized through HALO DB-20 UV-absorption spectra with the wavelength range of 300-700 nm. Samples were sonicated for 30 min and the aqueous solution was subsequently evaluated at room temperature for the optical band gap. Crystalline nature of powdered samples was evaluated by X-ray diffraction analysis (Shimadzu-6000), operating at 30 kV and 40 mA. Elemental compositions and size of the synthesized NPs was characterized using SEM (SIGMA model, MIRA3 TESCAN) operated at an accelerating voltage of 10 kV and EDX detector. All the apprehensible functional groups involved in capping and reduction of ZnO NPs were identified using FTIR (Bruker V70).

### In vitro cytotoxicity screening against HepG2 cell line

#### Cell culture

Human hepatocellular carcinoma cells (ATTC HB-8065) were cultured in DMEM containing 10 % Fetal calf serum (FCS), supplemented with 2 mM L-glutamine, 1 mM Na-pyruvate, 100 U/mL penicillin, 100 μg/mL streptomycin at 37 °C in a humidified 5 % CO_2_ atmosphere. Cell harvesting was done with 0.5 mM trypsin/EDTA at room temperature for 1 min.

#### Cell viability assay

ZnONPs as well as CE and WPE were evaluated for their cytotoxic activity against HepG2 cell line through SRB assay as previously described by Arooj et al. (2015[8]). Nanoparticles were suspended in deionized water and extracts were dissolved in DMSO for cytotoxicity screening. HepG2 cells (> 90 % confluency) were seeded in a 96-well plate at a density of 12000 cells/well and allowed to adhere for 24 hrs at 37 °C. Subsequently, cells were treated with 200 µg/ml NPs as well as callus and plant extract for 24 hrs. Cells were fixed by adding 50 % pre-chilled TCA and incubated at 4 °C for 1 hr, followed by rinsing with deionized water thrice. The resulting plates were then air dried and cells were stained with 0.01 % SRB dye followed by incubation for 30 min at room temperature and washing with 1% acetic acid for the removal of unbounded dye. Untreated cells and DMSO were used as negative and doxorubicin (34 µM) was used as positive control in the experiment. Blanks representing background optical density consisted of sample only and media only controls. Photographs were taken using Olympus CK2 light microscope equipped with digital camera. Experiment was repeated twice with triplicates for each sample. SRB dye was solubilized by adding 100 µl of 10 mM Tris (pH 8) into each well at room temperature for 5 min. Absorbance values were analysed at 565 nm wavelength using Microplate reader (Platos R 496, AMP) (Supplementary Table 1). Percentage viability relative to untreated sample was calculated by using the following formula:


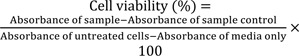


Percentage inhibition was calculated by formula:

Cell inhibition (%) = 100 - Cell viability (%).

### Microbial susceptibility test

Investigation of four multiple drug resistant bacterial strains i.e., *Bacillus subtilis *(ATCC 6051),* Pseudomonas aeruginosa *(ATCC 9027), *Klebsiella pneumoniae* (ATCC 70068) and *Staphylococcus epidermidis *(ATCC 12228) was carried out for biocidal activities of ZnONPs. Microbial susceptibility testing was performed by agar disc diffusion technique. Bacterial broth was evenly dispensed on nutrient agar plate at density of 1×10^8^ CFU/ml, previously refreshed in shaking incubator at 37 °C, 200 rpm in nutrient agar broth (Oxoid-CM0003). Zinc Acetate Dihydrate (2 mM) and Amoxicillin (standard antibiotic) (10 µg/ml) were added in the experiment as negative and positive controls respectively. Samples were dissolved in deionized water prior to experiment. Each sterile filter disc placed on culture plate, was loaded with 10 mg/ml of sample and incubated for 24 hrs at 37 °C. Zones of inhibition (mm) were measured afterwards. Antibacterial assay was performed in triplicates.

### Statistical analysis

All statistical measurements were performed in triplicates and analysed by Pareto analysis of variance (ANOVA) with Duncans multiple range test (Duncan, 1955[12]). Values in text and in figures were statistically analysed as mean ± SD (standard deviation). All the graphical analyses were done by using OriginPro software. The probability was considered to be significantly different at the level of P˂ 0.05 (95 %). 

## Results and Discussion

### Phytochemical investigation

A number of primary and secondary metabolites (phytochemicals) have been reported to perform primal role in the reduction of metal ions to NPs. The selection of plants for the purpose of NPs biosynthesis is mostly based on their capacity to produce important secondary metabolites that plays their respective role as capping and reducing agents. These capping and reducing agents stabilize the NPs, thus helping them to avoid agglomeration. Ponarulselvam et al. (2012[39]) selected* Catharanthus roseus* for the evaluation of vincristine and vinblastine as phyto-reducing agents. Both were selected due to their potential nematicidal activities. Similarly, Akhtar et al. (2016[6]), reported that plectranthoic acid inhibits proliferation of prostate cancer cells by the activation of 5′AMP-activated kinase (AMPK). Present study reveals the maximum production of TPC (11.3 mg g^-1^ DW) and TFC (0.54 mg g^-1^ DW) when treated with TDZ (2.0 mgL^-1^) in case of CE. The TPC and TFC for WPE were 6.1 mg and 0.5 mg g^-1^ DW, respectively (Figure 1B(Fig. 1)). Presence of sufficient amount of TPC and TFC shows that *Isodon rugosus *can prove efficient in ZnONPs biosynthesis.

### UV-Vis analysis of ZnO NPs

Optical characterization of the ZnONPs was carried out using UV-Vis absorption spectrophotometer in the range of 300-700 nm. Figure 1C(Fig. 1) shows characteristic spectra of ZnONPs synthesized using CE and WPE of *Isodon rugosus*. Dispersed ZnONPs absorbed the radiations approximately at 362 nm in case of CE mediated ZnONPs (C-ZnONPs) and 382 nm in case of WPE mediated ZnONPs (W-ZnONPs). Narrowing of the peaks confirms the reduction of zinc acetate into ZnONPs. Indramahalakshmi (2017[23]) and Jamdagni et al*.* (2016[24]) also reported similar characteristic peaks for ZnONPs. The band gap energy of C-ZnONPs and W-ZnONPs was 3.692 keV and 3.691 keV respectively, representing characteristic absorption peaks of ZnONPs. This may be due to the presence of different fractions of secondary metabolites in both CE and WPE, which in turn resulted in shape variation in ZnONPs. Similar findings were previously reported by Manokari et al. (2016[30]).

### XRD results

**Figure 2:**
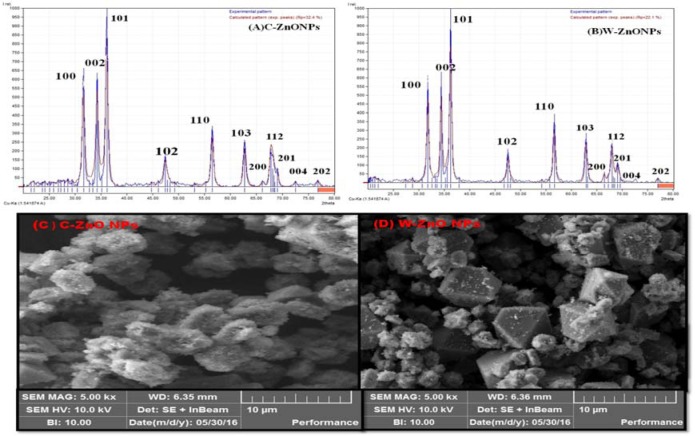
XRD and SEM analysis of ZnONPs where (A) XRD image of C-ZnONPs, (B) XRD image of W-ZnONPs, (C) SEM image of C-ZnONPs and (D) SEM image of W-ZnONPs Abbreviations: C-ZnONPs: callus derived naoparticles, W-ZnONPs whole plant derived nanoparticles

X-ray diffraction determines phase purity (crystalline nature), structural properties, thickness of thin films and the atomic arrangement of amorphous materials. In XRD, X-rays penetration provide information about overall structure of the material under observation. Diffraction pattern given in Figure 2A and 2B(Fig. 2) shows several peaks in the range of 30-80^°^ which corresponds to 100, 002, 101, 102, 110, 103, 200, 112, 201, 004 and 202 crystal planes, respectively. The data revealed crystalline hexagonal structure of ZnONPs.

### SEM and EDX results

SEM images of the biosynthesized ZnONPs are shown in Figure 2 (C, D(Fig. 2)). It is observed that alkaline pH results in shape-dependent synthesis of ZnONPs. Higher pH results in availability of large number of functional groups for capping and stabilization of NPs. The exact mechanism, however, is difficult to grasp due to involvement of large amounts of diverse phyto-reducing metabolites. Current study revealed that C-ZnONPs were irregular hexagonal shaped (Figure 2C(Fig. 2)) while W-ZnONPs were roughly triangular (Figure 2D(Fig. 2)). The stability and morphology of ZnONPs were increased by the combined effect of pH and reducing agents (primary and secondary metabolites of samples extract). Previous findings suggested that the adjustment of pH was compulsory for the stability of ZnONPs. The current findings are well supported by the findings of Song et al. (2009[46]) and Amin et al. (2012[7]). Ghodake et al. (2010[15]) reported similar findings that alkaline pH is responsible for hexagonal and triangular shaped NPs. Altering pH results in modification of the surface charge upon the secondary metabolites which alternatively affects their binding capacity and reduction potential of metal ions during the synthesis of NPs (Kumar and Yadav, 2008[28]; Pereira et al., 2015[37]). Sathishkumar et al*.* (2010[43]) confirmed that alkaline pH plays a major role in shape and size variations of NPs. Additionally, alterations in shape and size of NPs may also occur due to other parameters like reaction time, salt concentration, localization of NPs, and change in functional molecules.

**Figure 3:**
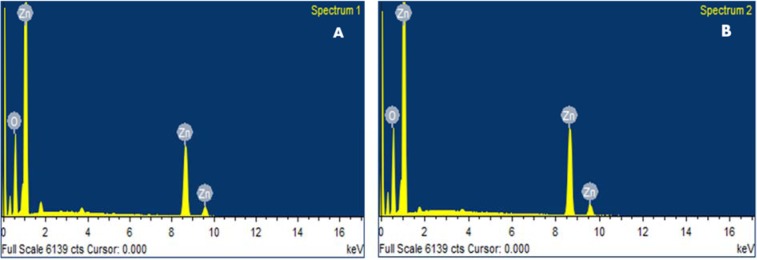
Figure 3: EDX analysis ZnONPs showing the purity of zinc and oxygen ions in the samples (A) C-ZnONPs, (B) W-ZnONPs

The chemical composition of ZnONPs was confirmed by EDX-ray spectrum. The EDX profile exhibits strong signals of zinc and oxygen in both C-ZnONPs and W-ZnONPs (Figure 3A, B(Fig. 3)), (Table 1(Tab. 1)).

### FTIR analysis

**Figure 4:**
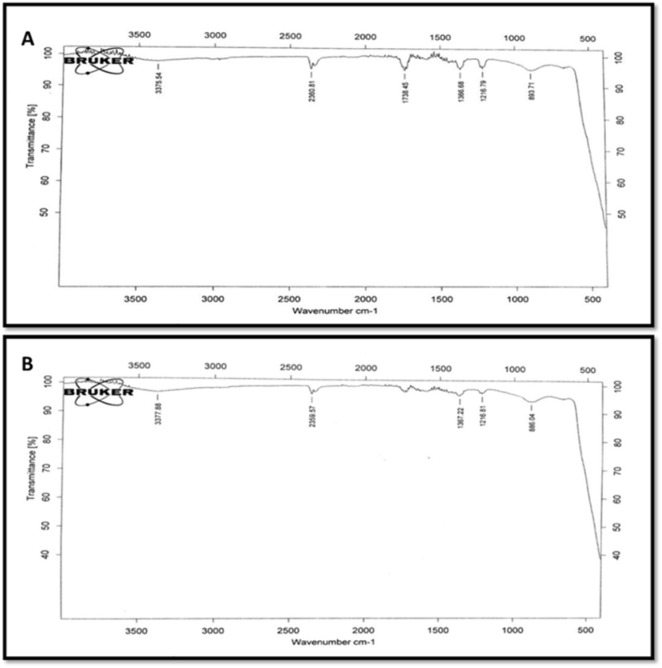
FTIR spectra of powdered samples of (A) C-ZnO NPs, (B) W-ZnO NPs

The FTIR spectra of ZnONPs (CE/WPE) was observed in the range of wave-numbers 500-3500 cm^-1^ to identify the functional groups involved in the reduction and capping/stabilization of the synthesized NPs (Figure 4(Fig. 4)). FTIR analysis confirmed the presence of Zn-O bond in both C-ZnONPs and W-ZnONPs due to the presence of peaks at 893.71 cm^−1^ and 886.04 cm^−1^, respectively (Pinto and Nazareth, 2016[38]). ZnONPs showed broad bands at 3375.54 cm^−1 ^(C-ZnONPs) and 3377.88 cm^−1 ^(W-ZnONPs) which was attributed to the stretching vibrations of hydrogen bond (O-H) as reported by Thirumavalavan et al. (2013[51]). Spectra at 1216.79 cm^−1^ (C-ZnONPs) and 1216.81 cm^−1 ^(W-ZnONPs) confirm C-N stretch bond of aliphatic amines. Peaks at 2360.81 cm^−1 ^(C-ZnONPs) and 2359.57 cm^−1 ^(W-ZnONPs) represent asymmetric C=O stretching (Shaikh et al*.* 2017[44]).

These findings clearly demonstrate the attachment of primary and secondary metabolites to the ZnONPs. Electrostatic forces between the negatively charged groups of organic molecules and positively charged zinc ions are responsible for the binding of these functional groups. This interaction makes these NPs a perfect candidate for various applications such as drug delivery (Wang et al., 2013[53]) and study of biological interactions (Song et al., 2009[46]). 

### In vitro cytotoxicity screening against HepG2 cell line

**Figure 5:**
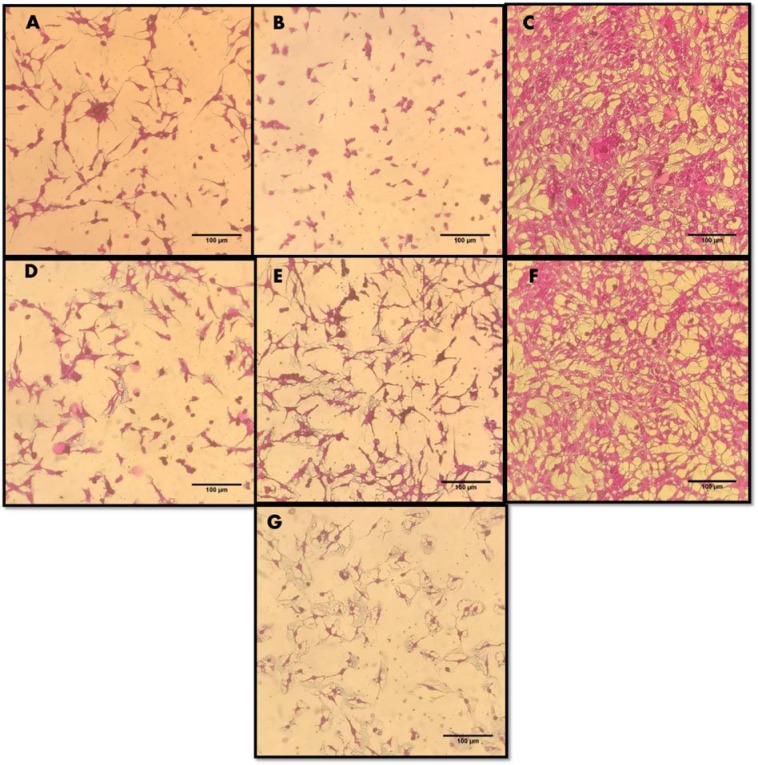
Cytotoxic effects of ZnONPs and extracts on HepG2 cells upon 24 hour treatment with 200 µg/ml concentration. Untreated cells, DMSO (solvent) and Doxorubicin (34 µM) were included as controls. Microscopic images of HepG2 cells (treated and untreated). Magnification = 200X, Scale = 100 µm. (A) Callus extracts (CE). (B) Callus derived ZnONPs (C-ZnONPs). (C) Untreated HepG2 cells. (D) Whole plant extracts (WPE). (E) Whole plant derived ZnONPs (W-ZnONPs). (F) DMSO 1% (negative control). (G) Doxorubicin 34 µM (positive control)

**Figure 6:**
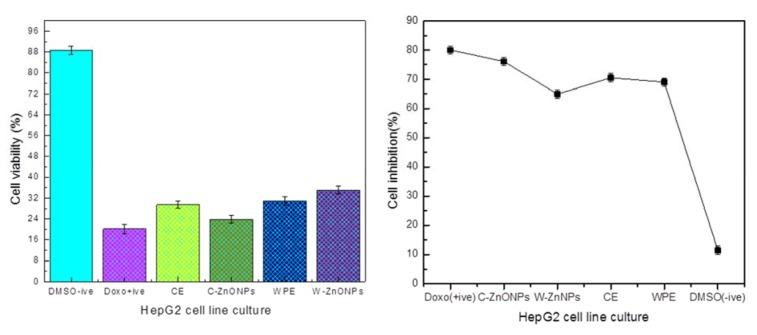
Percentage viabilities and inhibition of cells relative to untreated control (Mean ± SD). Each sample was studied in triplicates and experiment was performed twice.

Suforhodamine B assay was used to screen *in vitro* cytotoxicity of extracts and NPs using HepG2 cell line. Ionic strength and pH of NPs depend upon formation of hydroxyl (OH^−^) ions which directly influence the repulsion and attractions of particles (Peng et al., 2017[36]). Cells were treated with 200 µg/ml of ZnONPs (C-ZnONPs and W-ZnONPs), CE and WPE for 24 hours. Our results suggested that CE showed slightly higher cytotoxicity (29.47 %) as compared to WPE (31 %). Morphological changes were also observed in both samples i.e., cells became long and fibrous. On the other hand, cell viability in C-ZnONPs was lower (23.92 %) as compared to W-ZnONPs (35 %). Morphologically, cells became small and round upon treatment of C-ZnONPs while long and fibrous cells were observed in the presence of W-ZnONPs. Najim et al. (2014[33]) also reported morphological changes induced by cytotoxic ZnONPs on different cell lines (U937, SH-SY5Y, differentiated SH-SY5Y, and Hs888Lu). Morphological changes in cells may affect their metastatic process including substrate attachment, migration and invasion (Brandhagen et al., 2013[11]). DMSO (1 %), used as a negative control showed slight cytotoxicity and doxorubicin (positive control) showed high cytotoxicity at 34 µM concentration (Figures 5(Fig. 5), 6(Fig. 6)). 

**Figure 7:**
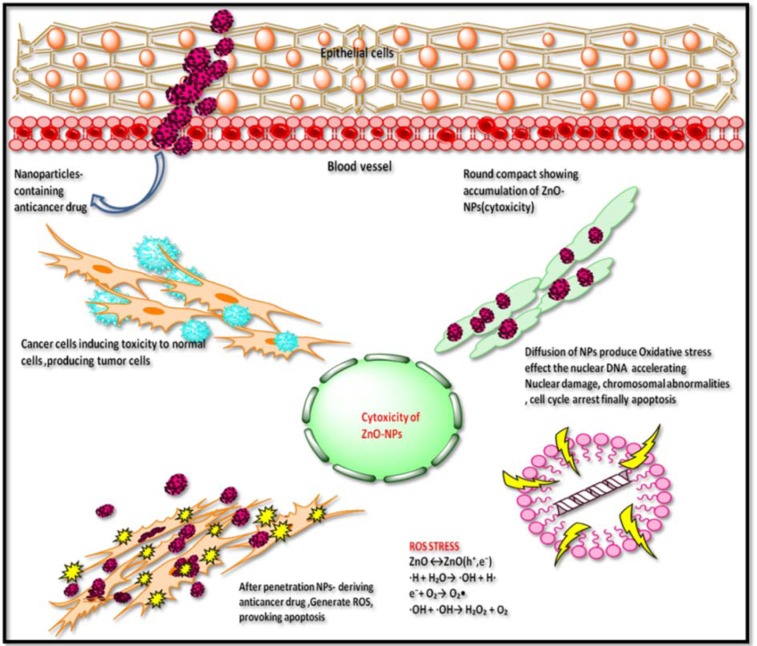
Tentative graphical representation of ZnONPs cytotoxicity towards HepG2 cell line. ZnONPs induce cytotoxicity by morphological changes, loss of membrane integrity, cell shrinkage, and reduced cell density which are characteristic features of apoptotic cell formation.

Akhtar et al. (2012[5]) reported cytotoxicity of ZnONPs in HepG2 cells due to production of reactive oxygen species. In spite of unique physical and chemical properties of metallic NPs, quantum size and shape also assist in the field of therapeutics (Khan et al., 2017[26]). In present study, the surface charge ratio of ZnONPs was pH dependent to absorb various chemical clusters (-ZnOH^2+^, -ZnOH, -ZnOˉ). In this regard, it was hypothesized that surface orientation of ZnONPs allows them to conjugate with protein targets or chemical groups. Therefore, ZnONPs possess cytotoxicity towards cancer cells (Figures 5(Fig. 5), 6(Fig. 6)). Literature suggests formation of ROS as leading cause of cell death by apoptosis (Figure 7(Fig. 7)).

Our present findings suggest configurational role of ZnONPs towards cytotoxicity. It was established that callus derived hexagonal NPs (C-ZnONPs) showed high cell inhibition (% viability 23.92 %) as compared to whole plant derived triangular NPs (W-ZnONPs) in which cell viability was 35 % (Supplementary Table 2). From FTIR and SEM data, we concluded that this may be due to difference in surface charge of secondary metabolites which eventually control the bonding of NPs with cancer cells. Suresh et al. (2018[49]) supported similar results. They reported that biologically synthesized hexagonal ZnONPs exhibit good cell inhibition and viability against Daltons Lymphoma Ascites (DLA). 

Also, *in vitro* derived callus extract (CE) showed cell viability of 29.47 % while whole plant extract (WPE) showed 31 % cell viability (Figure 6(Fig. 6)). The current findings are in agreement with the results of Kamalanathan and Natarajan (2018[25]). They reported that potency of callus was better than wild leaf extract sample against human breast cancer cell line MCF-7. Literature review proved presence of commercially important anticancer phenolics and triterpenoids i.e., plectranthoic acid, limonene, rosmarinic acid, caffeic acid (Abdel-Mogib et al., 2002[2]; Akhtar et al., 2016[6]; Hossan et al., 2014[20]; Hafidha et al., 2018[17]; Krishna et al., 2017[27]). To the best of our knowledge very little data is available that compares the cytotoxicity of CE, WPE, C-ZnONPs and W-ZnONPs against HepG2 cell line.

### Effect of ZnONPs on bactericidal activity 

**Figure 8:**
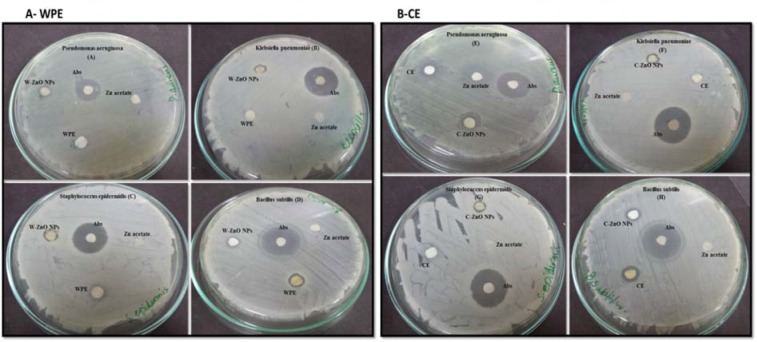
Antibacterial evaluation of Plant derived ZnONPs and extract (A-WPE) and Callus derived (B-CE) ZnONPs and extract analysed by disc diffusion method, showing zone of inhibition against two Gram-positive (*Staphylococcus epidermidis, Bacillus subtilis)* and two Gram-negative bacteria (*Klebsiella pneumoniae, Pseudomonas aeruginosa).* Zone of inhibition were measured in mm, taking sample concentration 10 mg/mL. Notes: Values (mean ± SD) indicates of three experiments Abbreviations: Abs, amoxicillin antibiotic; WPE, whole plant extract; W-ZnONPs, whole plant derived zinc oxide nanoparticles; CE, callus extract; C-ZnONPs, callus derived ZnONPs; Zn acetate, zinc acetate dihydrate salt; SD, standard deviation

Literature reported the increased emergence of drug resistant bacteria in the clinical field directs the scientific community to look for alternatives of antibiotics to minimize the risk from bacterial infections. Essential oils produced from *Isodon rugosus *consist of complex mixture of volatile molecules such as sesquiterpene, amorphene, germacrene-D, triterpenoids, plectranthoic acid A and B, acetyl plectranthoic acid and plectranthadiol. These volatiles compounds potentially exhibit bactericidal property against various human pathogenic bacteria. Similarly Hussain et al. (2017[22]) and Faleiro (2011[14]) reported the effectiveness of essential oils against human pathogenic bacteria. The bactericidal action of essential oils is linked to its hydrophobicity, resulting in increased cell permeability and subsequent leakage of cell constituents. Permeability of ZnONPs and their capability to bind with the cell membrane generates reactive oxygen species (ROS), thus enhancing the efficacy against drug-resistant bacterial strains (Abinaya et al., 2017[4]). Our results (Figure 8A*,* B(Fig. 8)) were in accordance with the data reported by Premanathan et al. (2011[40]). According to them, the effects of ZnONPs were more potent against Gram-positive bacterial strains as compared to Gram-negative strains. Our evaluation of potency of ZnONPs also revealed more potent bactericidal activity against Gram-positive *S. epidermidis *and *B. subtilis* as compared to Gram-negative*, K. pneumoniae* and *P. aeruginosa*, respectively (Table 2(Tab. 2)).

It was hypothesized that Gram-positive bacteria possess specific cell wall components that help in embedding ZnONPs within the cellular wall. The presence of ZnONPs within the walls results in cellular disintegration and leakage of internal components. In case of Gram-negative bacteria, no such embedding is observed hence only a small amount of surface bound ZnONPs causes partial leakage of internal contents.

**Figure 9:**
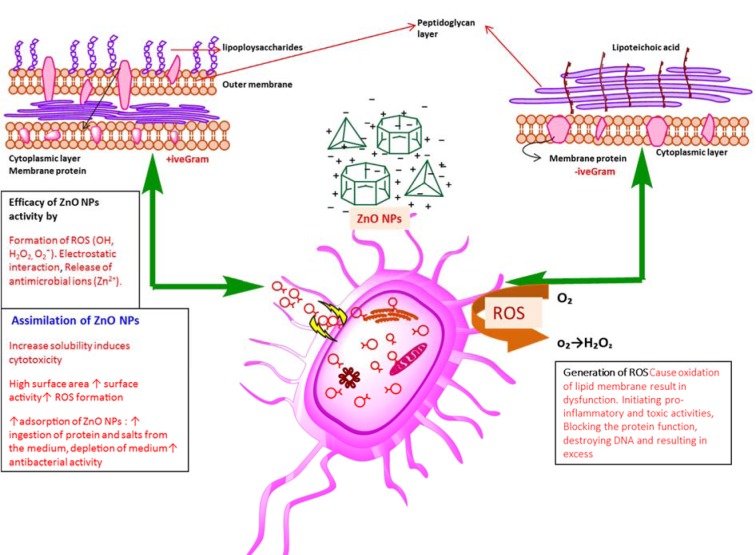
Diagrammatic representation of ZnONPs cytotoxicity towards human pathogenic bacterial strains depending upon many factors such as shape (transformation of particles as function of pH), production of ROS, dysfunction of bacterial membrane, effect of oxidative stress on DNA, protein, mitochondria (cell death), electrostatic forces, charge surface area, ultimately causing death of microbes

Our findings endorse the idea of biocidal effect of ZnONPs due to combination of various mechanisms such as ROS production in response to zinc ions within the bacteria. Minor sensitivity of *S. epidermidis,* *B. subtilis *compared to* K. pneumoniae* and *P. aeruginosa* was due to fact that the outer layer of Gram-positive bacteria is tightly packed due to assembly of lipopolysaccharide (LPS) and peptidoglycans. Furthermore, rational estimation of decline in sensitivity may be due to reduced stability of O_2_**^¯^** materialization at the surface and the decrease of pH value in the medium (Tang and Lv, 2014[50]) (Figure 9(Fig. 9)).

## Conclusions

Our results conclude that *Isodon rugosus *possesses essential bioactive compounds responsible for therapeutic properties of the plant against microbial and cancer diseases. The FTIR analysis revealed that capping of ZnONPs was due to the phenolics, flavonoids, amino acids and amides that were collectively involved in stabilizing ZnONPs. SEM and EDX confirmed the hexagonal and triangular shapes of C-ZnONPs and W-ZnONPs, respectively. The hexagonal C-ZnONPs were more effective against Gram-positive bacterial strains as compared to Gram-negative bacterial strains, while triangular W-ZnONPs were found less effective. The biocompatibility, stability and morphology of ZnONPs generally depend upon the pH at which they were synthesized. Cytotoxicity of ZnONPs against HepG2 cell lines was found to be morphology and surface chemistry dependent. Based on enhanced biocompatibility, improved solubility and less toxicity, the efficacy of ZnONPs synthesized from *Isodon rugosus* in the field of medicine could play a significant role in future. In the light of current research, ZnONPs could be developed as a potential next generation cancer treatment strategy. In order to do so, further research must be carried out in order to resolve the issue of cancer, a long-term health hazard.

## Compliance with ethical research standard

All the experimental work reported in this article was performed in accordance with principles of ethical research that complies with all applicable legislation. All the authors certify that there is no conflict of interest over any content of this article.

## Supplementary Material

Supplementary data

## Figures and Tables

**Table 1:**
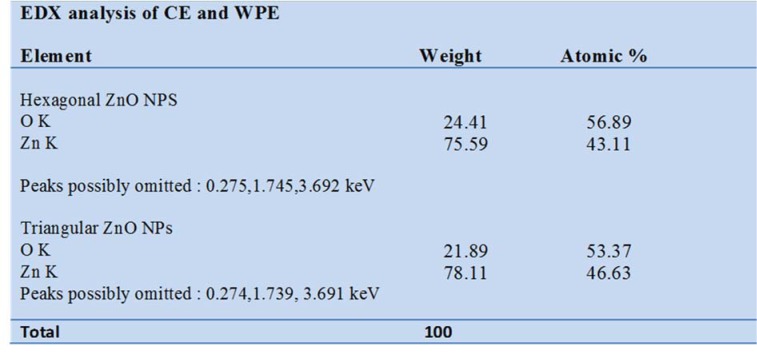
Atomic percentage of zinc and oxygen in ZnONPs as obtained from EDX analysis

**Table 2:**
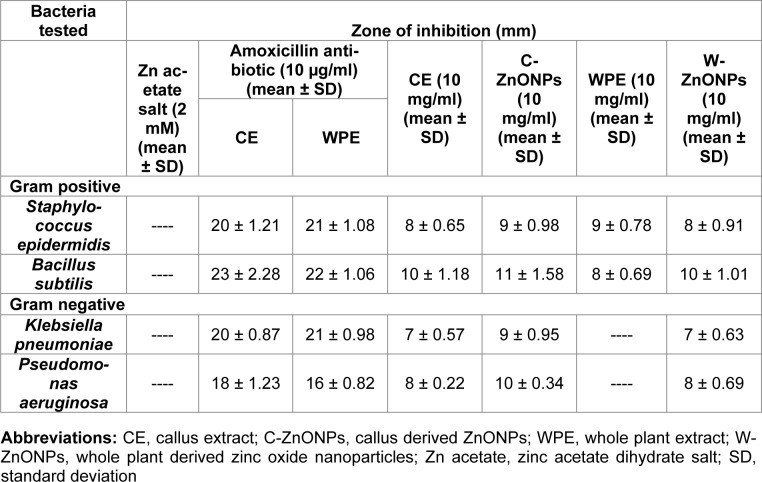
Antibacterial activity of CE, WPE, C-ZnO NPs and W-ZnO NPs against two Gram-positive (*Staphylococcus epidermidis, Bacillus subtilis*) and two Gram-negative (*Klebsiella pneumoniae, Pseudomonas aeruginosa*) bacteria
