# Supplementary material for: Exploiting in vitro potential and characterization of surface modified Zinc oxide nanoparticles of Isodon rugosus extract: Their clinical potential towards HepG2 cell line and human pathogenic bacteria
Source: EXCLI J. 2018 Jul 16;17:671–87. doi: 10.17179/excli2018-1327 (PMC6088224; doi:10.17179/excli2018-1327)
Supplement: Supplementary data [file EXCLI-17-671-s-001.pdf]

EXCLI Journal 2018;17:671-687 – ISSN 1611-2156
Received: May 10, 2018, accepted: June 18, 2018, published: July 16, 2018

Supplementary data to:

EXPLOITING *IN VITRO* POTENTIAL AND CHARACTERIZATION OF SURFACE MODIFIED ZINC OXIDE NANOPARTICLES OF *ISODON RUGOSUS* EXTRACT: THEIR CLINICAL POTENTIAL TOWARDS HEPG2 CELL LINE AND HUMAN PATHOGENIC BACTERIA

Aisha Siddiquah[1], Syed Salman Hashmi[1], Sadaf Mushtaq[1], Sullivan Renouard[2], Jean Philippe Blondeau[3], Rashda Abbasi[4], Christophe Hano[2], Bilal Haider Abbasi[1,2]*

[1] Department of Biotechnology, Quaid-i-Azam University, Islamabad-45320, Pakistan
[2] Laboratoire de Biologie des Ligneux et des Grandes Cultures (LBLGC), UPRES EA 1207, Université d'Orléans, Chartres, France
[3] Conditions Extrêmes et Matériaux: Haute Température et Irradiation (CEMHTI) CNRS UPR3079, 1D avenue de la Recherche Scientifique, 45071 Orléans, France
[4] Institute of Biomedical & Genetic Engineering (IBGE), Sector G-9/1, Islamabad, Pakistan

* Corresponding author: Bilal Haider Abbasi, Tel & Fax: 0092-51-90644121, Mobile: 0033-767-97-0619, E-mail: bhabbasi@qau.edu.pk

http://dx.doi.org/10.17179/excli2018-1327

This is an Open Access article distributed under the terms of the Creative Commons Attribution License (http://creativecommons.org/licenses/by/4.0/).

EXCLI Journal 2018;17:671-687 – ISSN 1611-2156
Received: May 10, 2018, accepted: June 18, 2018, published: July 16, 2018

Supplementary Table 1: Protocol for SRB Assay

SRB Assay				
**Cell seeding**	**Treatment**	**Cell fixation**	**Cell Staining**	**Dye Solubility**
1200 cells/well in 96 well plate (100 ul/well)	Done for 24 hours at 37°C, 200µg/ml of samples (1µl/well)	Addition of 50% TCA (25µl/well) at 4°C for 1 hours followed by washing	Addition of 0.01% SRB dye (50µl/well) for 30min at room temperature followed by washing with 1% acetic acid	Addition of 10mM Tris (pH8) to dissolve SRB dye(100 µl/well) for 5min, reading taken at 565nm

Abbreviations: TCA: Trichloroacetic acid, Tris: tromethamine; SRB: Sulforhodamine B

Supplementary Table 2: Raw data for cytotoxicity tests. Percentage cell viability and inhibition of HepG2 cell line

Results		
**Samples**	**% viability (mean)**	**% inhibition (mean)**
**CE**	29.47 ±1.4	70.53 ± 1.48
**C-ZnO NPs**	23.92 ± 1.5	76.08 ±1.44
**WPE**	30.98 ± 1.6	69.02 ± 1.47
**W-ZnO NPs**	35.12 ±1.4	65 ± 1.45
**DMSO**	88.6 ± 1.7	11.4 ± 1.5
**Doxorubicin**	20.11± 1.8	80 ± 1.46

Abbreviations: C-ZnO NPs: Callus derived zinc oxide nanoparticles; W-ZnO NPs: Whole plant derived zinc oxide nanoparticles; HepG2 Cell Line Hepatocellular carcinoma cells; CE: Callus extract; WPE: Whole plant extract; DMSO: Dimethyl sulfoxide (negative control); Doxorubicin: positive control
